# Damage to intestinal barrier integrity in piglets caused by porcine reproductive and respiratory syndrome virus infection

**DOI:** 10.1186/s13567-021-00965-3

**Published:** 2021-06-23

**Authors:** Jin Zhao, Shuangxiu Wan, Na Sun, Panpan Sun, Yaogui Sun, Ajab Khan, Jianhua Guo, Xiaozhong Zheng, Kuohai Fan, Wei Yin, Hongquan Li

**Affiliations:** 1 College of Veterinary Medicine, Shanxi Agricultural University, Taigu, 030801 Shanxi China; 2 School of Pharmacy, Heze University, Heze, 274000 Shandong China; 3 Laboratory Animal Center, Shanxi Agricultural University, Taigu, 030801 Shanxi China; 4 Department of Veterinary Pathobiology, Schubot Exotic Bird Health Center, Texas A&M University, College Station, TX 77843 USA; 5 Centre for Inflammation Research, Queen’s Medical Research Institute, The University of Edinburgh, Edinburgh, EH16 4TJ UK

**Keywords:** Porcine reproductive and respiratory syndrome (PRRS), Lung-gut axis, Intestinal barrier function, Inflammatory response

## Abstract

Porcine reproductive and respiratory syndrome (PRRS) induces respiratory disease and reproductive failure accompanied by gastroenteritis-like symptoms. The mechanism of intestinal barrier injury caused by PRRSV infection in piglets has yet to be investigated. An in vivo PRRSV-induced model was established in 30-day-old piglets by the intramuscular injection of 2 mL of 10^4^ TCID_50_/mL PRRSV for 15 days. Observations of PRRSV replication and histology were conducted in the lungs and intestine, and goblet cell counts, relative MUC2 mRNA expression, and tight junction protein, proinflammatory cytokine, TLR4, MyD88, IκB and p-IκB expression were measured. PRRSV replicated in the lungs and small intestine, as demonstrated by absolute RT-qPCR quantification, and the PRRSV N protein was detected in the lung interstitium and jejunal mucosa. PRRSV infection induced both lung and gut injury, markedly decreased villus height and the villus to crypt ratio in the small intestine, and obviously increased the number of goblet cells and the relative expression of MUC2 mRNA in the jejunum. PRRSV infection aggravated the morphological depletion of tight junction proteins and increased IL-1β, IL-6, IL-8 and TNF-α expression by activating the NF-κB signalling pathway in the jejunum. PRRSV infection impaired intestinal integrity by damaging physical and immune barriers in the intestine by inducing inflammation, which may be related to the regulation of the gut-lung axis. This study also provides a new hypothesis regarding the pathogenesis of PRRSV-induced diarrhoea.

## Introduction

Porcine reproductive and respiratory syndrome (PRRS) is a major immunosuppressive disease caused by porcine reproductive and respiratory syndrome virus (PRRSV) that threatens the pig industry worldwide. The major clinical characteristics of PRRS include respiratory disorders and reproductive failure, usually accompanied by gastroenteritis-like symptoms, such as diarrhoea and haematochezia. PRRSV coinfection with pig epidemic diarrhoea virus (PEDV) not only impairs intestinal morphology but also decreases intestinal barrier integrity in growing pigs [1]. PRRSV infection reduces villus height in the cranial, medial, and caudal segments of the small intestine [2].

The intestinal mucosal barrier plays an important role as the first barrier against pathogens in defending against harmful external factors and maintaining intestinal health. Impaired intestinal function caused by pathogens will lead to obstructed nutritional absorption [3], low immunity [4], and reduced growth performance of pigs [5–7], causing severe economic losses to the pig industry. Epithelial cells and their tight junctions create a barrier between the external environment and the host. Tight junctions are multiprotein complexes composed of transmembrane proteins and peripheral membrane proteins [8]. Tight junction proteins commonly include zonula occludens (ZO), occludin and the claudin family proteins. Tight junction proteins limit intestinal epithelial cell permeability and protect mucosal cells from being exposed to bacteria and toxic macromolecules.

The intestinal mucosal surface is covered by a hydrated gel formed by mucins, which create a barrier that prevents large particles (mostly bacteria) from directly contacting the epithelial cell layer. Mucins are secreted by goblet cells. Goblet cells differentiate from pluripotent stem cells whose origin is the base of the intestinal crypt [9]. Goblet cells are a specific type of secretory cell that synthesizes and secretes various factors, trefoil peptides and mucins, which together form an intestinal mucus layer to protect intestinal epithelial cells [10]. The secreted products of goblet cells play an important role in innate immunity during intestinal infections [11].

The NF-κB pathway is the main signalling pathway involved in secondary immune responses to various stimuli and the main pathway involved in intestinal inflammation [12, 13]. These proinflammatory factors contribute to the stimulation of protective immune responses, leading to the development of excessive systemic inflammatory reactions and causing inflammatory lesions [14–16]. The activation of the NF-κB pathway and the secretion of proinflammatory cytokines are important indicators of gut inflammation and immune barrier dysfunction.

Previous studies indicated that PRRSV infection impairs the lungs and intestine [17] and affects jejunal function and integrity due to hypophagia [18]. However, its impact on intestinal barrier function and the correspond mechanism of intestinal barrier injury remain unclear. In this study, the impact of PRRSV infection on intestinal barrier function and the associated mechanism of intestinal barrier injury in pigs were evaluated by measuring PRRSV replication in tissue, the expression of tight junction proteins, the effects on goblet cells, and the expression of TLR4 MyD88, IκB, p-IκB and proinflammatory cytokines in the jejunum.

## Materials and methods

### Animals and inoculations

Landrace piglets at approximately 30 days of age with a weight of 10.12 ± 3.04 kg were used in this study. The piglets were seronegative for PRRSV antibodies at the beginning of the experiment according to a commercial PRRSV antibody rapid test card (Combined, China) and were also confirmed to be free of PRRSV, porcine circovirus type 2 (PCV2), porcine parvovirus, pseudorabies virus, swine influenza virus and PEDV by the PCR testing of serum.

Six piglets were randomly divided into two groups: a PRRSV-infected group and a control group (*n* = 3 in each group). The animals were raised at the experimental animal centre of Shanxi Agriculture University (Jinzhong, Shanxi, China) and were housed at 25 ℃-28 ℃ in single pens. The pigs were fed a common corn-soybean nursery diet and had free access to food and water. All animal work adhered to the standards of ethical and humane use of animals for research. Pigs in the PRRSV-infected group were intramuscularly injected with 2 mL of a 10^4^ TCID_50_/mL PRRSV isolate suspension (JS-1, provided by the Jiangsu Academy of Agricultural Sciences, passaged in Marc-145 cells), and pigs in the control group were simultaneously injected with DMEM culture medium. The experimental pigs were clinically examined to assess their general behaviour and appetite and the presence or absence of clinical signs of respiratory disease or diarrhoea each day from 0 to 15 days post-infection (dpi). At 15 dpi, the pigs were euthanized by the intravenous injection of an overdose of xylazine hydrochloride.

### Pathological and immunohistochemical analyses

Tissue samples from the lungs and segments of the duodenum, jejunum and ileum of 6 pigs were fixed in Bouin’s fixative and 10% neutral buffered formalin (for Alcian Blue staining). Paraffin sections were prepared via dehydration, clearance, embedding and sectioning steps. The haematoxylin and eosin (H&E) protocol (Solarbio, Beijing, China) was performed to visualize pathological changes in lung and intestine tissue samples. The periodic acid Schiff (PAS) method (MXB, Fuzhou, China) and Alcian blue staining (pH = 2.5, Solarbio, Beijing, China) were performed to visualize the count of neutral and acidic mucin-secreting goblet cells per villus or crypt in the jejunum according to the manufacturer’s instructions. Photomicrographs of the abovementioned sections were taken with a microscope with an attached camera (Olympus, Japan). The images contained at least 8 villus and crypt pairs per section, and villus height and crypt depth were measured with Image-Pro Plus 6.0 (Media Cybernetics, Bethesda, MD, USA). The measured data are presented as the means with the standard errors of the means.

Villi atrophy, fusion, and inflammatory infiltration were assessed by veterinary pathologists blinded to the treatment groups. A score of 0, 1, 2 or 3 was assigned to assess the severity of villus atrophy, fusion and inflammatory infiltration. A score of 0 indicated no pathological or morphological changes; 1 indicated mild villus atrophy, fusion and light inflammatory infiltration; 2 indicated moderate villus atrophy and fusion with moderate inflammatory infiltration; and 3 indicated severe villus atrophy and fusion with severe inflammatory infiltration.

The sectioning, deparaffinizing and rehydration of jejunum tissue samples and slides were performed in the same way as in the H&E staining procedure. Immunochemistry staining was carried out with the SP Rabbit HRP Kit (DAB) following the manufacturer’s protocol (CWBIO, China). After blocking with a normal sheep serum working solution, the tissue sections were incubated overnight at 4 ℃ with a rabbit anti-ZO-1 polyclonal antibody (1:100, Bioss, Beijing, China), a rabbit anti-occludin polyclonal antibody (1:200, Proteintech, China) or a rabbit anti-claudin 1 polyclonal antibody (1:200, Proteintech, China). The DAB-treated sections were stained with haematoxylin, dehydrated, cleared, and sealed with neutral gum. The average optical density of intestinal tight junction proteins was quantified using ImageJ software. ImageJ was used to measure the optical density and the positive area of each villus. The average optical density (AOD) was calculated using the optical density per unit area of each villus in each image. Three villi were chosen in each slice.

### Immunofluorescence assay

Lung and jejunum tissue slides were deparaffinized and rehydrated in the same way as in the H&E staining procedure. To enhance immunoreactivity, antigen retrieval was performed by incubating the slides in a 1 × sodium citrate antigen retrieval solution (Solarbio, Beijing, China) for 30 min at 95 ℃. After cooling to room temperature, the slides were washed with PBS for 5 min. The sections were permeabilized with 0.2% Triton for 5 min, blocked with 5% normal goat serum for 20 min and incubated with a PRRSV N protein antibody (1:100, Bioss, Beijing, China) at 4 ℃ overnight, after which they were incubated with FITC-conjugated goat anti-rabbit IgG (1:100, Proteintech, China) at room temperature for 1 h. The specimens were covered with mounting medium and then with a cover slip, followed by fluorescence visualization using an electric fluorescence microscope (Olympus, Japan).

### Quantitative real-time PCR

Total RNA was extracted from lung, duodenum, jejunum and ileum tissue samples from pigs according to the TRIzol protocol (Invitrogen, Carlsbad, CA, USA). The RNA concentration and purity were evaluated using an Eppendorf BioPhotometer D30 (Eppendorf, USA). The extracted RNA samples with an OD260/280 between 1.8 and 2.0 and an OD260/230 between 1.7 and 2.0 could be used for subsequent experiments. Complementary DNA was synthesized with the PrimeScript® RT Master Mix kit with gDNA Eraser (TaKaRa, Dalian, China) according to the manufacturer’s protocol. RT-qPCR was performed by using a 7500 Real-Time PCR System (ABI, USA). Relative RT-qPCR was performed to quantify the mRNA expression of tight junction proteins and proinflammatory cytokines using 2 × SYBR Green qPCR Master Mix (Low ROX, Biotool, USA). Relative expression levels were determined with the 2^−∆∆Ct^ method. Absolute RT-qPCR was conducted to quantify PRRSV N gene expression, and a standard curve was generated using a serially diluted plasmid containing the N gene. The primers used for RT-qPCR are presented in Table 1.

**Table 1:** **Primers used for quantitative real-time PCR**

Gene name	Sense (5’-3’)-forward	Antisense (5’-3’)-reverse
GAPDH	TCGGAGTGAACGGATTTGGC	TGACAAGCTTCCCGTTCTCC
ZO1	AGGCGTGTTTAACAGCAACG	CCAAAGGACTCAGCAGGGTT
OCLN	TCGTCCAACGGGAAAGTGAA	ATCAGTGGAAGTTCCTGAACCA
CLDN1	TGGAAGATTTACTCCTACGCTGG	TCCCAGCAGGATGCCAATTACC
MUC2	GGACGCCTACAAGGAGTTCG	ACCAGCTGCTGAGTGAGGTA
PRRSV N Gene	AGAAGCCCCATTTCCCTCTA	CGGATCAGACGCACAGTATG
IL-1β	CCCAAAAGTTACCCGAAGAGG	TCTGCTTGAGAGGTGCTGATG
IL-6	ACAAAGCCACCACCCCTAAC	CGTGGACGGCATCAATCTCA
IL-8	TTCACAAGTCTCTGCTCAACTG	TGTCCTCAAGGTAGGATGGG
TNF-α	CCCTCACGTCCTTCTGGTTT	GAGTCTGGAAGCCCCAGTTC

### Western blotting assay

Jejunum proteins were prepared using a RIPA lysis kit (Solarbio, Beijing, China). The protein concentration was examined via the bicinchoninic acid (BCA) method. Equivalent amounts of total protein were separated by sodium dodecyl sulphate–polyacrylamide gel electrophoresis (SDS-PAGE) and then transferred to polyvinylidene fluoride membranes. The membranes were blocked in Tris-buffered saline and Tween 20 (TBST) containing 5% bovine serum albumin (BSA) for 2 h at room temperature and incubated first with primary antibodies against GAPDH (1:10 000, Proteintech, China), TLR4 (1:500, Proteintech, China), MyD88 (1:1000, Proteintech, China), IκB (1:2000, Proteintech, China), p-IκB (1:1000, Cell Signaling Technology, USA), IL-1β (1:1000, Thermo Fisher, USA), IL-6 (1:500, Thermo Fisher, USA), IL-8 (1:600, R&D System, USA), and TNF-α (1:500, R&D System, USA) at 4 ℃ overnight and then with HRP-conjugated goat anti-mouse, goat anti-rabbit or donkey anti-goat IgG for 2 h at room temperature. The membranes were subsequently washed with TBST, and the immunoreactive proteins were visualized using an enhanced chemiluminescent detection kit (CWBIO, China). The densitometric values of protein bands were quantified by using Image-Pro Plus 6.0.

### Data analysis

The data were analysed using GraphPad Prism 5.0 software (San Diego, CA, USA). The data are represented as the means of replicate samples and expressed as the mean ± standard error of the mean (SEM). Unpaired t-tests and one-way ANOVA were carried out for statistical analysis. *p* values below 0.05 were considered statistically significant.

## Results

### PRRSV infection causes pathological injury of the lungs and intestine

**Figure 1:**
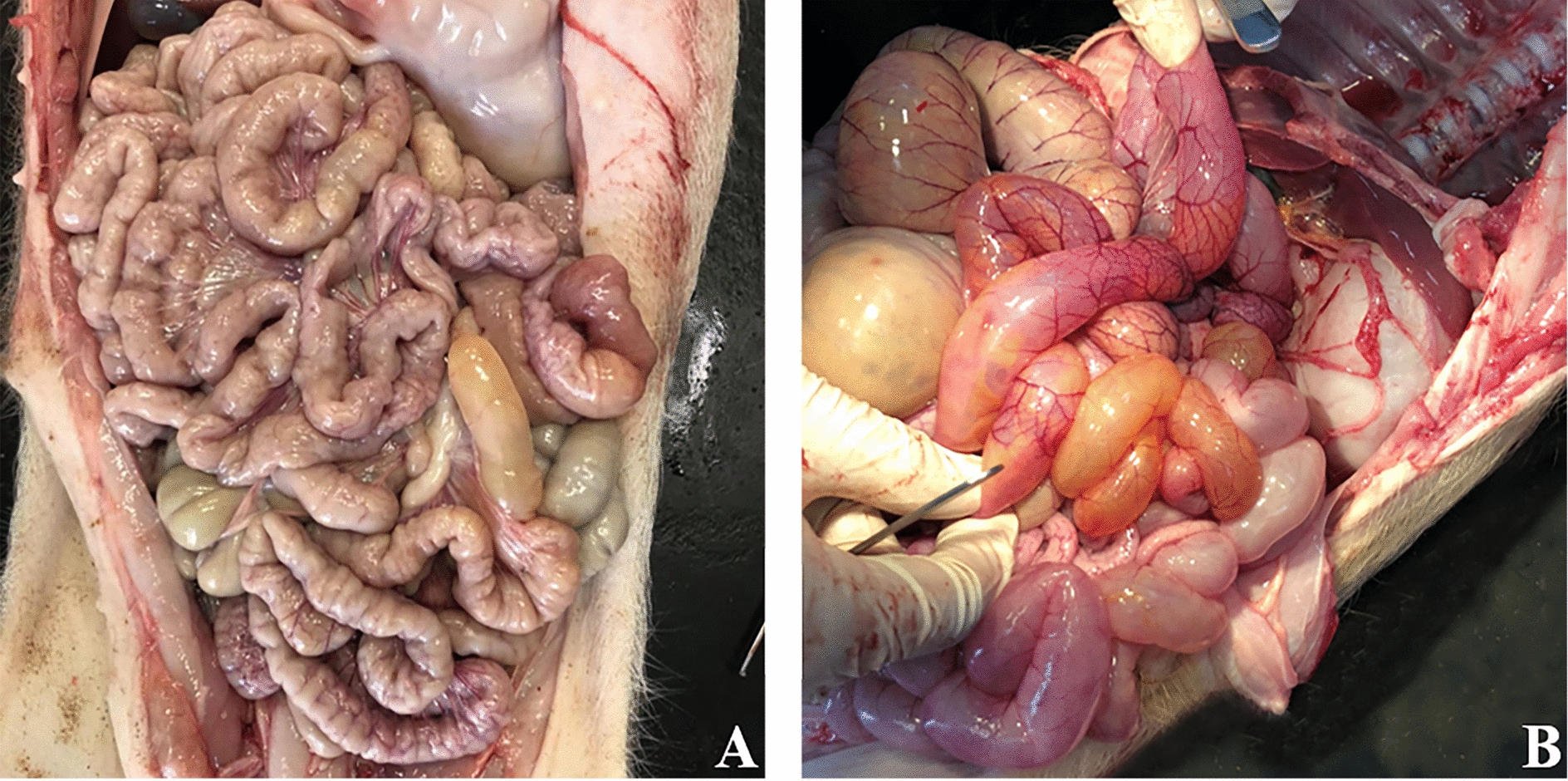
**Representative images of the intestine in the experimental pigs.** **A** control group. **B** PRRSV-infected group.

Piglets in the PRRSV treatment group showed progressive emaciation, declining food intake, depression, abdominal breathing, wheezing, coughing and gastroenteritis-like symptoms, such as vomiting and diarrhoea, at 7–15 dpi. All of these symptoms were obvious until the experiment was terminated. In addition, erythema and needle-like bleeding spots were observed behind the ears of PRRSV-infected pigs. In contrast, the piglets in the control group showed normal eating and drinking behaviour without any clinical symptoms throughout the study. The piglets in the PRRSV-infected group showed a markedly expanded intestinal track with watery yellowish contents, swelling and congestion (Figure 1B).

### PRRSV replicates in the lungs and intestine

**Figure 2:**
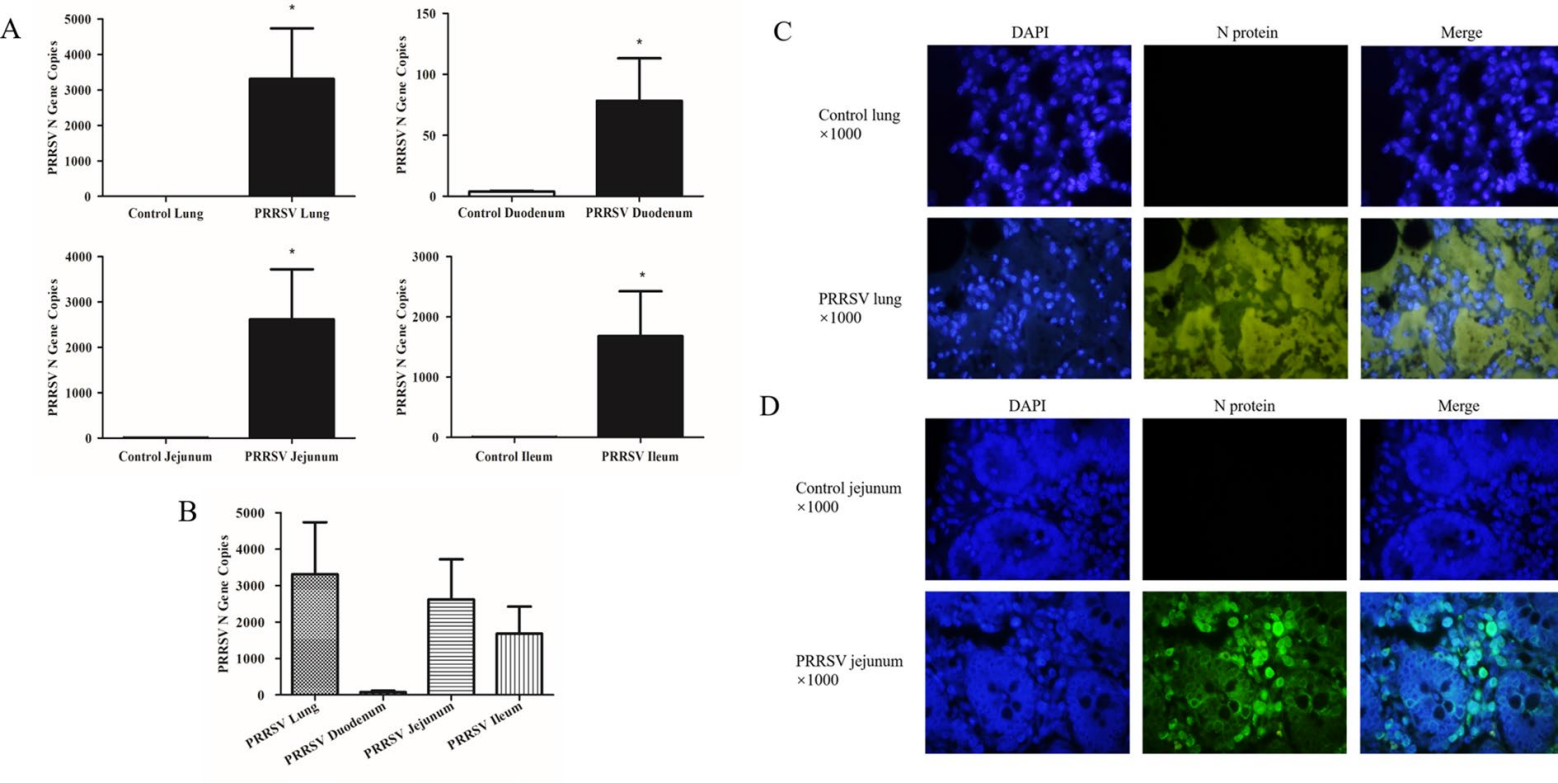
**Distribution of PRRSV in different tissue samples according to absolute RT-qPCR assays (A, B) and immunofluorescence analysis (C, D).** **A** PRRSV N gene copies in lung and intestine tissue samples of control and PRRSV-infected pigs. **B** Comparison of PRRSV N gene copies in lung and intestine tissue samples of PRRSV-infected pigs. **C** PRRSV N protein distribution in the lungs of control and PRRSV-infected pigs. **D** PRRSV N protein distribution in the jejunum of control and PRRSV-infected pigs. Data are expressed as the mean ± SEM. * *p* < 0.05, ** *p* < 0.01, *** *p* < 0.001.

The nucleocapsid protein (N) is the most highly expressed protein of PRRSV, and its expression is positively correlated with the copy numbers of PRRSV [19]. Absolute RT-qPCR was used to quantify the expression of the PRRSV N gene in lung and intestinal tissue samples collected at 15 dpi (Figures 2A and B). Compared with the control group, N gene expression in the lung and small intestine of the PRRSV-infected group was significantly increased (Figure 2A). The comparison of N gene copies in the lung and intestine tissues of the PRRSV-infected group showed that the N gene was mainly transcribed in the lungs and jejunum, but not at a significant level (Figure 2B). Immunofluorescence analysis showed that the N protein was expressed in both the lungs and jejunum of PRRSV-infected pigs. The N protein was mainly expressed in the interstitium of the lung (Figure 2C) and the intestinal mucosa of the jejunum (Figure 2D).

### PRRSV infection damages the lungs and the integrity of the small intestine

**Figure 3:**
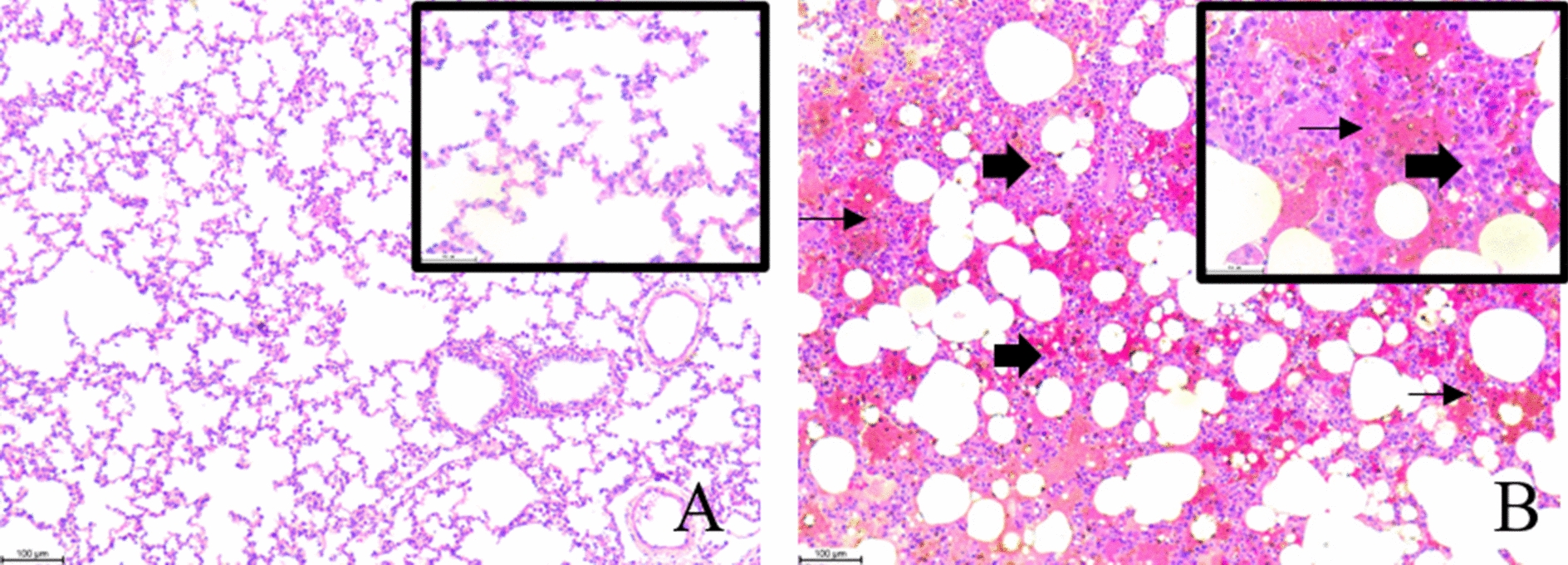
**Histopathological changes in lung.** Optical microscopy magnification: 100 × and 400 × . Scale bars: 100 μm and 50 μm. **A** control pigs. **B** PRRSV-infected pigs.

Severe lung and intestinal injury was observed after PRRSV infection. PRRSV-infected pigs showed severe monocyte and lymphocyte infiltration (thick arrow), thickened alveolar walls and exudates of inflammatory cells and erythrocytes in the alveolar space (thin arrow) (Figure 3).

**Figure 4:**
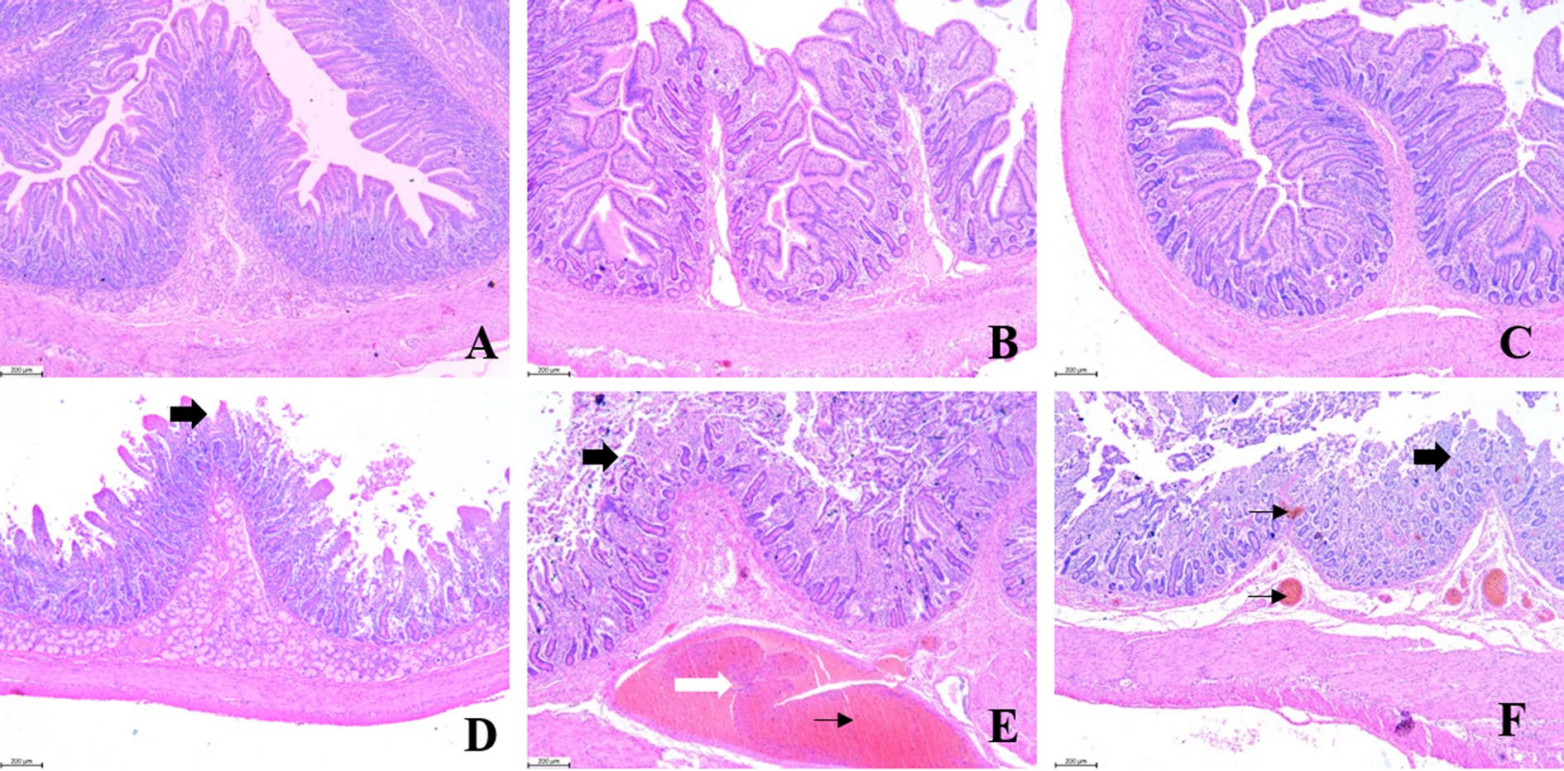
**Histopathological changes in the intestine.** Microscopy magnification: 50 × . Scale bar: 200 μm. **A**–**C** Duodenum, jejunum and ileum samples collected from control pigs. **D**–**F** Duodenum, jejunum, and ileum samples collected from PRRSV-infected pigs.

Villus height, crypt depth, and the ratio of villus height to crypt depth were determined in the duodenum, jejunum and ileum at 15 dpi (Figure 4; Table 2). Compared with the control group, the mucosal layers of PRRSV-infected pigs were significantly thinner, and the pathological symptoms in the PRRSV-infected group were more serious than those in the control group with regard to serious hyperemia of the mucosal and submucosal lamina propria (thin black arrow), infiltration of inflammatory cells in the mucosal lamina propria (thick white arrow), and atrophy and rupture of mucosal villi (thick black arrow). Necrosis and shedding of mucosal epithelial cells appeared in the PRRSV-infected group. PRRSV infection significantly reduced villus height in the duodenum (*p* < 0.001), jejunum (*p* < 0.001) and ileum (*p* < 0.001) but did not markedly affect crypt depth in all segments of the intestine, resulting in a decreased ratio of villus height to crypt depth; the decrease in the ratio was significant in the duodenum (*p* = 0.0027), jejunum (*p* < 0.001) and ileum (*p* < 0.001).

**Table 2:** **Effect of PRRSV infec﻿tion on intestinal pathology and clinical scores**

Parameter	Control group	PRRSV-infected group	*P* value
Duodenum			
Villus height (μm)	658.1 ± 26.75	459.2 ± 30.79^***^	< 0.001
Crypt depth (μm)	342.4 ± 15.06	339.3 ± 14.04	0.8817
Villus: crypt ratio	1.96 ± 0.12	1.38 ± 0.11^**^	0.0027
Lesion score	0.00	1.67 ± 0.75^**^	0.0041
Jejunum			
Villus height (μm)	567.13 ± 24.80	268.40 ± 19.58^***^	< 0.001
Crypt depth (μm)	252.35 ± 22.42	271.27 ± 14.89	0.4913
Villus: crypt ratio	2.39 ± 0.22	1.01 ± 0.10^***^	< 0.001
Lesion score	0.00	2.67 ± 0.47^***^	< 0.001
Ileum			
Villus height (μm)	499.80 ± 15.86	287.30 ± 14.04^***^	< 0.001
Crypt depth (μm)	238.01 ± 15.43	234.40 ± 8.13	0.8364
Villus: crypt ratio	2.15 ± 0.10	1.24 ± 0.07^***^	< 0.001
Lesion score	0.00	2.50 ± 0.50^***^	< 0.001

Data are expressed as the mean ± SEM. * *p* < 0.05, ** *p* < 0.01, *** *p* < 0.001.

Lesion scores showed significant differences in all segments of the two groups. Lesion scores in the duodenum, jejunum and ileum were notably increased in the PRRSV-infected group. The lesion score in the jejunum of PRRSV-infected pigs was highest (*p* < 0.001), suggesting deleterious effects of PRRSV infection in the jejunum.

### PRRSV infection increases goblet cell numbers and MUC2 expression in the jejunum

**Figure 5:**
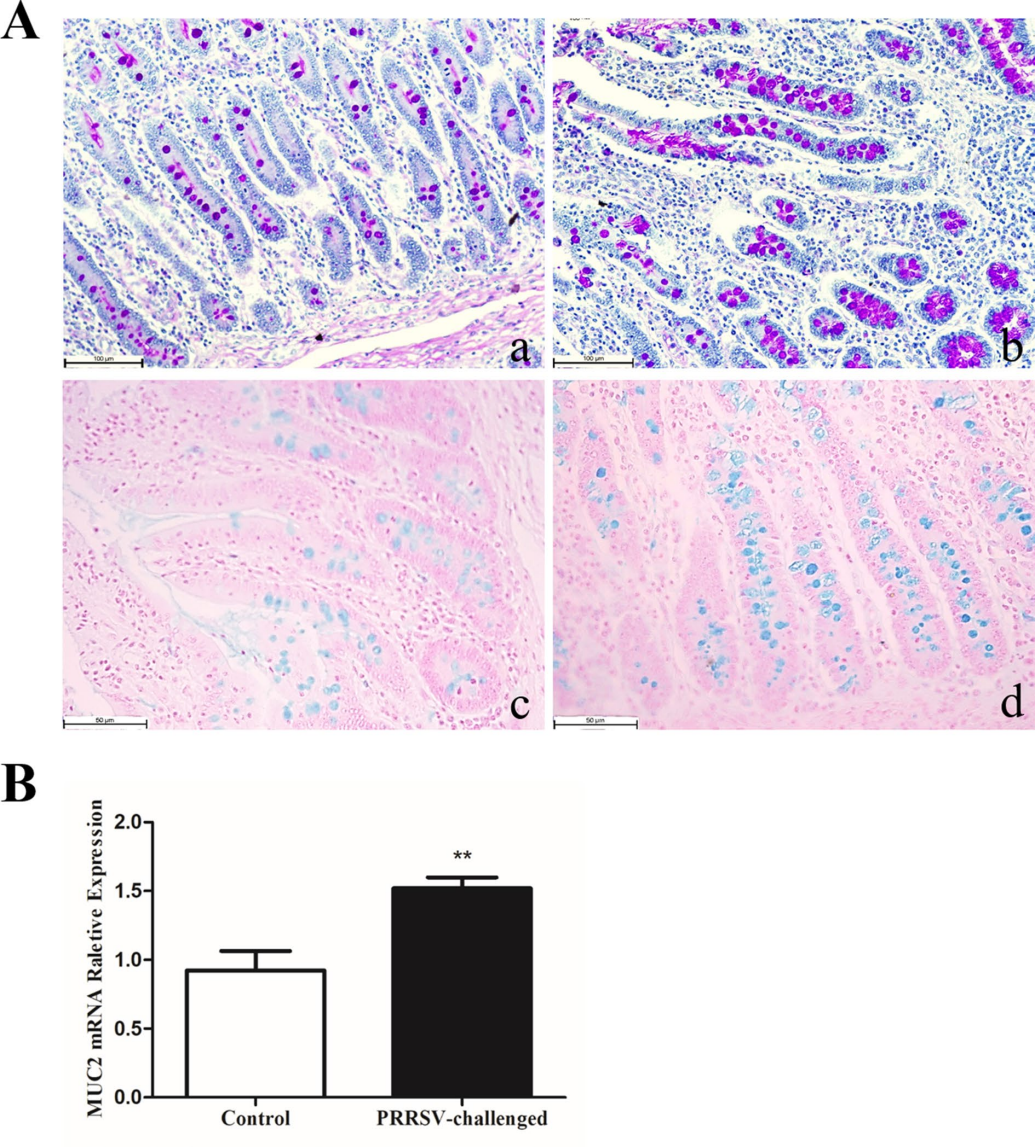
**Goblet cell numbers were detected using PAS and AB staining assays**. Microscopy magnification in a and b: 200 × . Scale bar: 100 μm. Microscopy magnification in c and d: 400 × . Scale bar: 50 μm. **A** PAS-stained goblet cells (a, b) and AB-stained goblet cells (c, d). a and c Jejunum of control pigs, b and d jejunum of PRRSV-infected pigs. **B** Relative MUC2 mRNA expression detected by relative RT-qPCR. Data are expressed as the mean ± SEM. * *p* < 0.05, ** *p* < 0.01, *** *p* < 0.001.

PAS and AB staining was carried out to detect the effect of PRRSV infection on the neutral and acidic mucin-secreting goblet cells of the jejunum. In infected pigs, the mean number of PAS (neutral mucin)-stained goblet cells per crypt in the jejunum was significantly (*p* < 0.001) higher than that in the control group. Similarly, the mean numbers of AB (acidic mucin)-stained goblet cells per crypt in the jejunum were significantly increased (*p* < 0.001). However, the mean numbers of AB-stained goblet cells in the jejunal villus were significantly reduced (*p* < 0.01) (Figure 5A; Table 3). This might be caused by shortening of villus height. Relative RT-qPCR analysis showed that relative MUC2 mRNA expression was increased by PRRSV infection (*p* < 0.01) (Figure 5B), suggesting that PRRSV infection activated goblet cells secreting mucins to defend against PRRSV.

**Table 3:** **Numbers of periodic acid Schiff (neutral mucin)- and Alcian blue (acidic mucin)-stained goblet cells per villus or crypt in the jejunum**

	Jejunal villus	Jejunal crypt
PAS-stained goblet cells		
Control	7.75 ± 0.55	5.92 ± 0.74
PRRSV-infected	7.67 ± 0.55	22.92 ± 2.30^***^
AB-stained goblet cells		
Control	9.58 ± 0.89	9.58 ± 0.88
PRRSV-infected	6.25 ± 0.63^**^	22.08 ± 1.30^***^

Data are expressed as the mean ± SEM. * *p* < 0.05, ** *p* < 0.01, *** *p* < 0.001 (statistically significant differences between the PRRSV-infected and uninfected pigs by the t-test).

### PRRSV infection decreases the expression of tight junction proteins in the jejunum

Intestinal permeability and epithelial tight junction barrier function play critical roles in the pathogenesis of diarrhoea [20]. To investigate the damage to intestinal permeability caused by PRRSV, the relative mRNA expression of tight junction proteins and the IHC staining of tight junction proteins in jejunum tissue samples were assessed.

**Figure 6:**
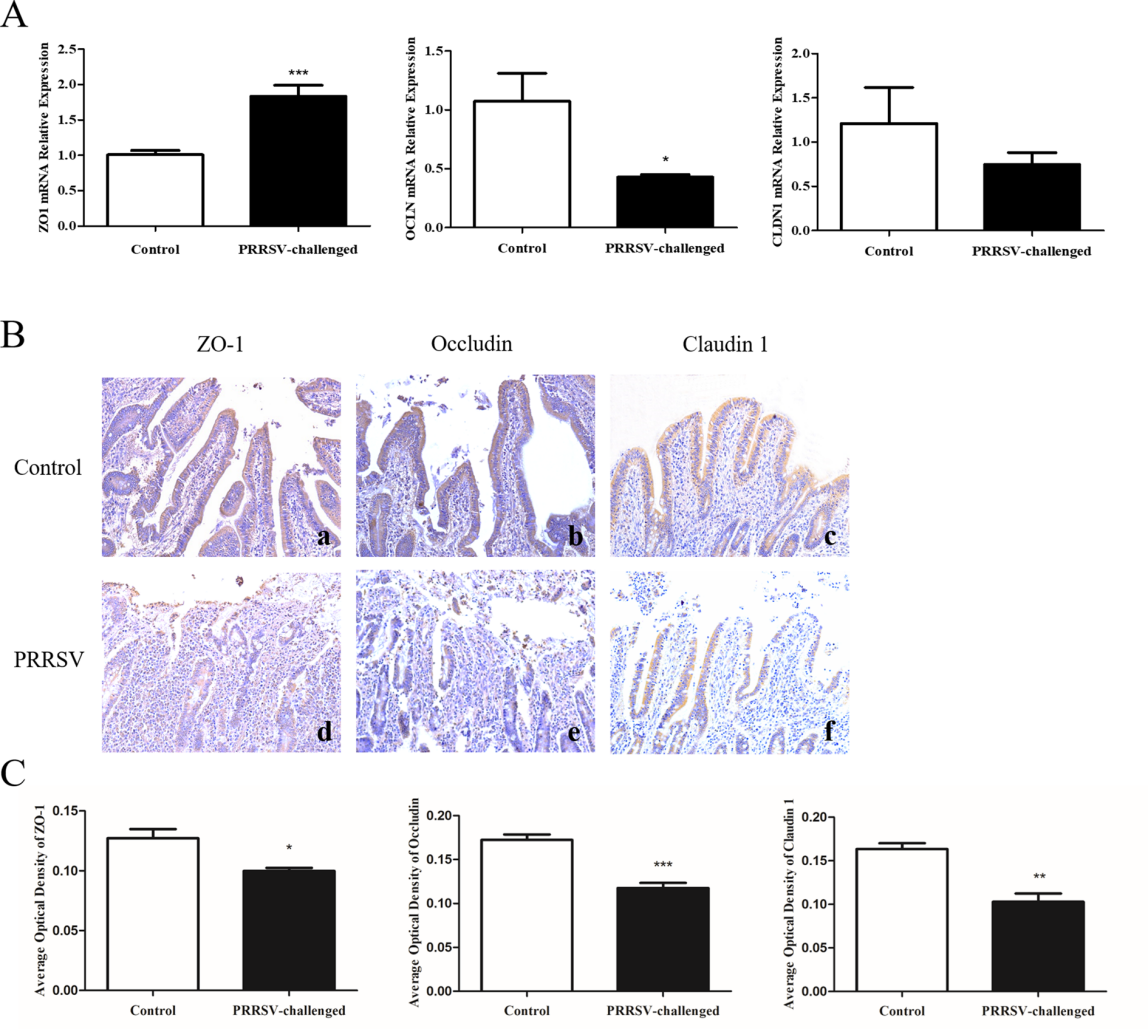
**Effect of PRRSV on the expression of tight junction proteins**. **A** Relative mRNA expression determined by relative RT-qPCR assays. **B** Representative histological sections of the jejunum. Tight junction protein expression was detected in the jejunum using IHC analysis. Microscopy magnification: 200 × . a, d ZO-1 expression, b, e Occludin expression, c, f Claudin 1 expression in the jejunum of control and PRRSV-infected pigs. **C** Average optical density of tight junction protein expression. All data are expressed as the mean ± SEM. * *p* < 0.05, ** *p* < 0.01, *** *p* < 0.00.

Interestingly, ZO1 expression was significantly increased by PRRSV infection compared with that in the control group (*p* < 0.001). OCLN was reduced by PRRSV treatment (*p* < 0.05). CLDN1 levels showed no difference between the control and PRRSV treatment groups (*p* > 0.05) (Figure 6A).

IHC staining and the analysis of the average optical density revealed that PRRSV treatment resulted in the significant depletion of ZO-1 (Figure 6B, panels a and d), Occludin (Figure 6B, panels b and e) and Claudin 1 (Figure 6B, panels c and f) in the epithelial junctions in the jejunum. Average optical density analysis showed that the ZO-1 protein level in the jejunum was lower in the PRRSV-infected pigs than in the control pigs (*p* < 0.05); occludin was significantly depleted in the jejunum of the PRRSV-infected pigs (*p* < 0.001); and the claudin 1 protein content was reduced in the jejunum of pigs in the PRRSV-infected group (*p* < 0.01) (Figure 6C).

### PRRSV infection activates the NF-κB signalling pathway in the jejunum

**Figure 7:**
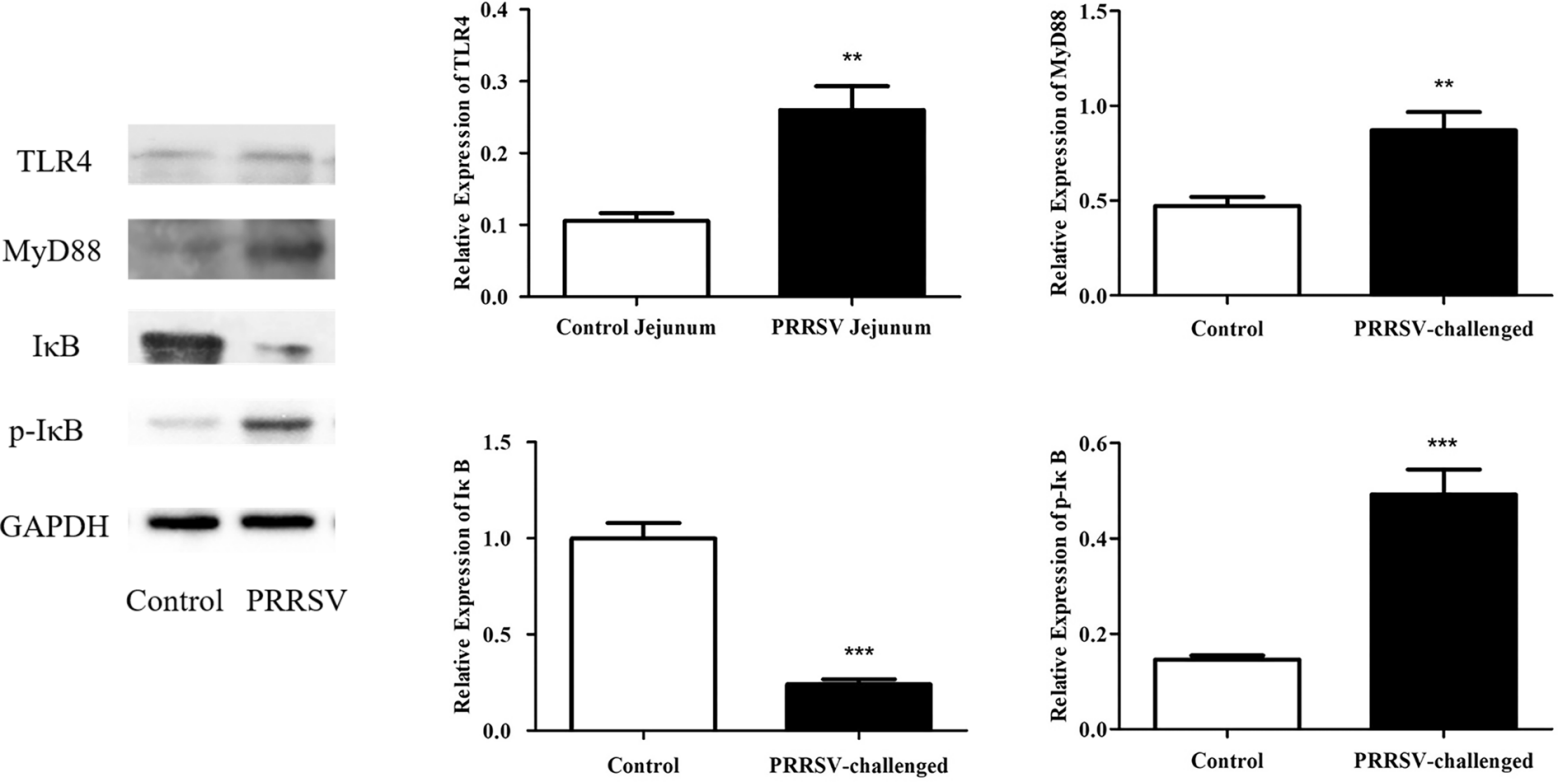
**Levels of TLR4, MyD88, IκB and p-IκB in jejunum tissue samples were detected using Western blotting.** Data are expressed as the mean ± SEM. * *p* < 0.05, ** *p* < 0.01, *** *p* < 0.001.

NF-κB is present in a non-activated state in the cytoplasm and combines with IκB (inhibitor of NF-κB) to form a complex. When an upstream signal that binds to the corresponding receptor on the membrane (usually mediated by myeloid differentiation factor 88 (MyD88)) and activates IκB kinase (IKK), IκB phosphorylation results in complex dissociation. This enables NF-κB to migrate to the nucleus and bind to the corresponding inflammation-related genes to initiate the transcription of inflammatory cytokines and induce inflammation. Therefore, the assessment of the expression of TLR4, MyD88, IκB and p-IκB is necessary to determine whether NF-κB is activated. To better understand the mechanism of PRRSV infection, we measured the expression of TLR4, MyD88, IκB and p-IκB in the jejunum at 15 dpi using Western blotting analysis. Western blotting showed that PRRSV infection resulted in a significant reduction in the expression of IκB (*p* < 0.001) and increases in the expression of p-IκB (*p* < 0.001), MyD88 (*p* < 0.01) and TLR4 (*p* < 0.01) (Figure 7).

### PRRSV infection promotes the expression of inflammatory cytokines in the jejunum

**Figure 8:**
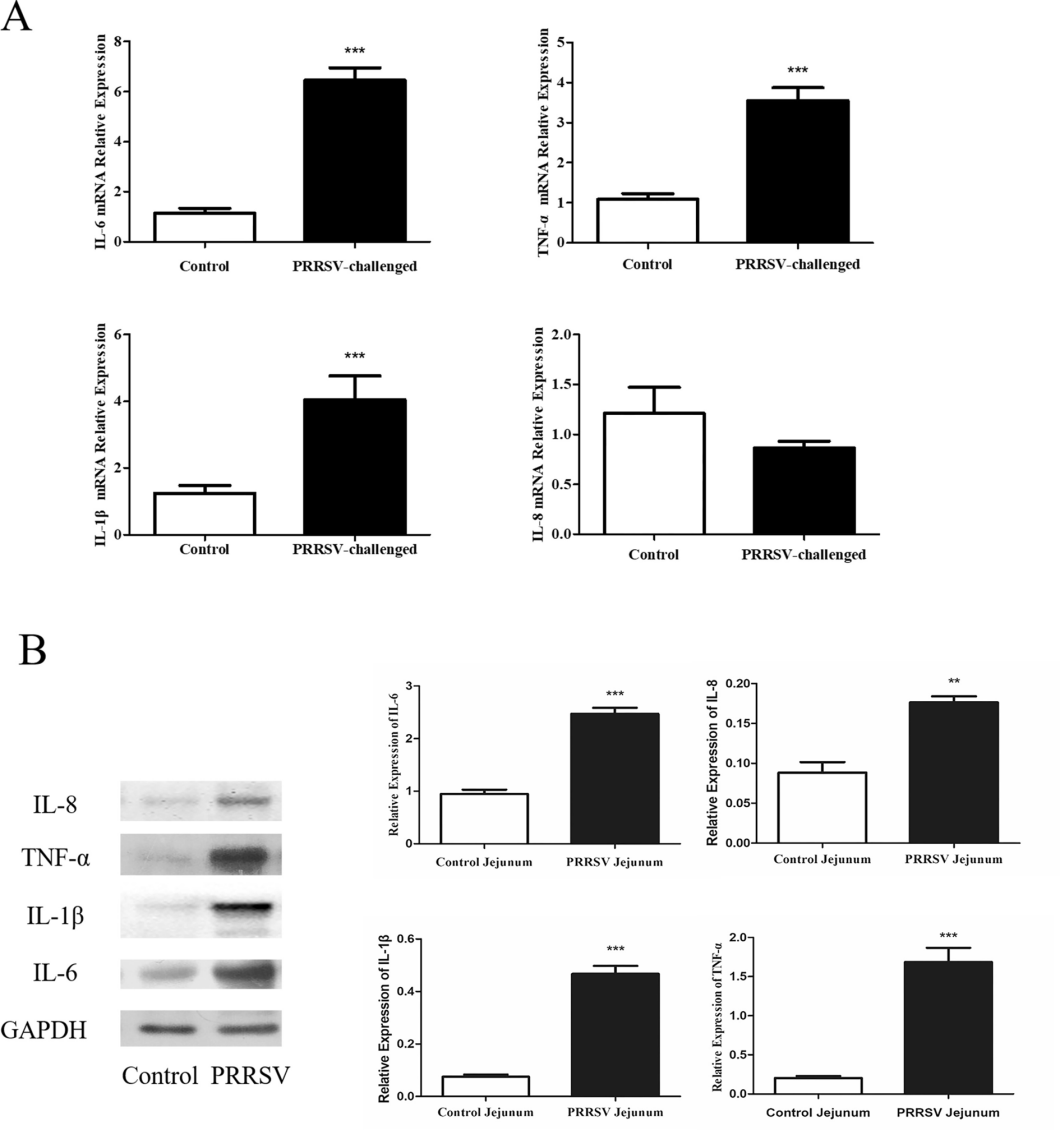
**Effect of PRRSV on the expression of proinflammatory cytokines.** **A** relative RT-qPCR assay. **B** Western blotting. Data are expressed as the mean ± SEM. * *p* < 0.05, ** *p* < 0.01, *** *p* < 0.001.

To investigate whether PRRSV has the ability to induce inflammatory cytokine production in the intestine, jejunum tissue samples were collected for relative RT-qPCR and Western blotting assays. The relative mRNA expression of IL-6 (*p* < 0.001), IL-1β (*p* < 0.001), and TNF-α (*p* < 0.001) was obviously increased in PRRSV-infected pigs (Figure 8A). In addition, treatment with PRRSV significantly induced the expression of IL-6 (*p* < 0.001), IL-1β (*p* < 0.001), TNF-α (*p* < 0.001) and IL-8 (*p* < 0.01) in jejunal tissue samples (Figure 8B).

## Discussion

Porcine reproductive and respiratory syndrome (PRRS) was identified in Europe in 1991 and emerged in the United States in 1992 [21, 22]; PRRS is a severe disease induced by porcine reproductive and respiratory syndrome virus (PRRSV). PRRSV infection causes severe animal morbidity and mortality, resulting in economic losses worldwide. Clinically, common characteristics of PRRS are lung injury [23] and reproductive failure [24], accompanied by gastroenteritis-like symptoms such as vomiting and diarrhoea [17].

An increasing number of studies have shown interactions between the lungs and gut in immunology. The gut and lungs possess a common origin in embryonic development involving in the common mucosal system [25–28]. It has also been reported that the intestinal microenvironment changes in the course of several different lung diseases, such as influenza virus infection and porcine circovirus type II infection [29–31]. Jiang et al. [32] demonstrated that Campylobacter and Clostridium became the two most abundant bacteria in the mucosal and luminal microbiota of the caecum of PRRSV-infected pigs, and their relative abundance was four times higher than that in healthy pigs, suggesting that Campylobacter and Clostridium might be associated with the pathogenesis of diarrhoea in PRRS. Our study showed that PRRSV replicated in the lungs and intestine, especially in the jejunum, indicating that the lungs and gut were involved in immune injury. The healthy intestinal mucosa hosts one of the largest populations of macrophages in the body [33]. PRRSV targets macrophages [34], and the variation of the PRRSV load may thus be due to the different numbers of macrophages in intestinal segments.

Interestingly, our study revealed that both the lungs and intestine were damaged in PRRSV-infected pigs. PRRSV-infected pigs showed respiratory symptoms such as abdominal breathing, wheezing, and coughing and gastroenteritis-like symptoms such as vomiting and diarrhoea. Microscopic pathological analysis showed pulmonary and intestinal injury. Histopathologic findings showed that PRRSV-infected pigs exhibited severe infiltration of monocytes and lymphocytes in the interstitium and thickened alveolar walls and that their intestinal integrity was seriously impaired with regard to atrophy and rupture of the mucosal villi and hyperemia of the mucosal and submucosal lamina propria. The ratio of villus height to crypt depth (V/C) reflects the digestive and absorption functions of the intestine. A decrease in V/C indicated a reduction in the digestive and absorption function of the intestine. Reductions in villus height and crypt depth in the cranial, medial, and caudal small intestinal segments have previously reported been in PRRSV-infected pigs [2]. Consistent with these findings, our study indicated that PRRSV infection decreased intestinal villus height and the V/C ratio, which contrasts with a previous report from Schweer et al. [1], who reported that PRRSV infection alone did not impact intestinal morphology. The differences between this study and the previous report are likely due to multiple factors, such as animal age, genetics and the timing of sampling.

A recent study demonstrated that epithelial barrier integrity is necessary to maintain “homeostatic tolerance” in response to physiological host-gut microbiome cross-talk [35]. Therefore, it is crucial to maintain an intact gut barrier in weaning pigs. Intact intestinal epithelial cells and tight junctions form the first line of defence in the intestinal barrier. Enterocytes express tight junction proteins, contributing to an intact, functional gut barrier to prevent toxins and microbial antigens from passing through the lamina propria [36]. Tight junctions are multiprotein complexes composed of transmembrane proteins, peripheral membrane proteins and regulatory molecules that include kinases [8]. The most important transmembrane proteins are the members of the claudin family, which determine junctional permeability. Occludin is a transmembrane tight junction protein that directly interacts with Claudins and actin and plays a role in barrier regulation and tumour suppression. Peripheral membrane proteins, such as zonula occludens 1 (ZO-1) and ZO-2, are crucial for tight junction assembly and maintenance.

A previous study reported that TNF-α induction increased intestinal epithelial tight junction permeability and could be considered an important proinflammatory mechanism contributing to intestinal inflammation in Crohn’s disease and other inflammatory conditions [37, 38]. TNF-α activates the NF-κB p65 signalling pathway and subsequently increases tight junction permeability [39]. TNF-α can reshape cytoskeletal microfilaments through the MLCK-MLC signalling pathway, leading to the contraction and tonicity of the actomyosin ring and causing the redistribution and destruction of tight junction proteins. Furthermore, it can induce the production of a variety of proinflammatory cytokines, such as IL-6 and IL-8, forming a cascade amplification effect, causing more damage to the intestinal mucosa [40–42]. In our study, TNF-α was significantly induced by PRRSV infection in the jejunum. The results of immunohistochemical experiments revealed that the expression of the tight junction proteins ZO-1, Occludin and Claudin 1 in the jejunum of the virus-infected group was significantly depleted relative to that in the normal group. A similar change was observed in the relative mRNA expression of OCLN and CLDN1 in the jejunum. The divergence between ZO1 mRNA and ZO-1 protein levels suggested that posttranscriptional and posttranslational regulation may occur between the ZO1 mRNA and the ZO-1 protein. In addition, in studies of biological adaptation to disease, it has been found that mRNA expression alone may not explain the kind of changes observed at the protein function level. This study suggested that PRRSV induced the disruption of intestinal barrier function by weakening tight junction barrier integrity.

The regulation of proinflammatory cytokine expression in the intestine involves NF-κB and MAPK signalling activation [43, 44]. NF-κB is a key transcription factor that regulates the activation of inflammatory cytokines and can be activated by virus infection, viral gene expression or LPS stimulation [45, 46]. An active NF-κB signalling pathway is a general prerequisite for the influenza virus infection of human cells, and sustained NF-κB expression is required for the activation of latent HIV-1 gene expression [47, 48]. IκB (inhibitor of NF-κB) combines with NF-κB p65 and p50 subunits in its inactive state. When the upstream signal (usually mediated by MyD88) activates IKK (IκB kinase), activated IKK phosphorylates and degrades IκB. The two subunits of NF-κB are activated from the inactivated state and transferred from the cytoplasm into the nucleus (especially the p65 subunit) to initiate the transcription of inflammatory cytokines and induce inflammation. In this study, PRRSV infection resulted in a significant increase in the expression of TLR4, MyD88 and p-IκB and a decrease in the expression of IκB in the jejunum, suggesting that PRRSV induced gut inflammation by activating the NF-κB signalling pathway. PRRSV infection has been shown to upregulate IL-1, IL-6, IL-8 and TNF-α levels in both in vivo and in vitro studies [49–51]. Our results showed that PRRSV significantly increased the protein expression of IL-1β, IL-6, TNF-α and IL-8 and the relative mRNA expression of IL-1β, IL-6, TNF-α in the jejunum compared with the results from control pigs, indicating that gut inflammation was successfully induced by PRRSV.

The gel-like mucus layer on the surface of the intestinal mucosa is formed by water, salt and mucins secreted by goblet cells. The gel-like mucus layer is part of the physical barrier of the intestine and protects intestinal epithelial cells from invasion by microbes in the intestinal cavity. The activation of the NF-κB pathway during intestinal inflammation causes an increase in MUC2 transcription [52, 53]. Mucins form a barrier to noxious substances in the intestinal lumina and are also a prerequisite for high concentrations of sIgA in the intestine [54]. Curry et al. [55] demonstrated that total acidic mucin levels were reduced in PEDV-infected pigs at 2 dpi and then increased compared with the levels in controls at 7 and 14 dpi. In our study, the number of acidic mucin-secreting goblet cells per crypt and MUC2 mRNA expression levels were evidently increased in PRRSV-infected pigs, and acidic mucins have been shown to be more resistant to bacterial degradation [56], suggesting that the body may compensate for defective intestinal integrity in the repair phase after PRRSV infection.

When the intestinal barrier is damaged, pathogenic intestinal viruses are exposed and transferred by M cells in the lymphoid follicle epithelium and subsequently presented to gut-associated lymphoid tissue (GALT) by dendritic cells; this activates the T subgroup in mesenteric lymph nodes (MLNs) and leads to the production of regulatory cytokines. Furthermore, the T cell subgroup in GALT and MLN can be recruited to the respiratory mucosa by CCR9, which acts as an immune homing molecule [57]. Therefore, the intestinal mucosal immune barrier, as the centre of the lung-gut axis, affects the immunity of both the lungs and intestine. The changes in innate and adaptive intestinal immune barriers after PRRSV infection need to be further explored.

Our current research focused on the intestinal barrier damage induced by porcine reproductive and respiratory syndrome virus and the underlying mechanism. The results showed that PRRSV infection aggravated lung and gut pathological injury by activating the NF-κB signalling pathway to induce the release of intestinal inflammatory cytokines, leading to injury to the integrity of the intestine and secretion of mucin. Considering our results and those of previous studies together, we speculate that the mechanism underlying the impact of PRRSV on the intestine is a complicated process involving the gut-lung axis. The interaction between PRRS and the homeostasis of the gut microbiota and how mucosal immunity is involved in this process need to be further explored. However, our findings will provide a new hypothesis regarding the pathogenesis of PRRSV-induced diarrhoea.

## Data Availability

The datasets used and analysed during the current study are available from the corresponding author upon reasonable request.
